# *“It’s a Godsend*”: Parental experiences of genomic testing for paediatric inborn errors of immunity

**DOI:** 10.1038/s41431-025-01917-7

**Published:** 2025-07-29

**Authors:** Amy Clark, Emily DeBortoli, Marisa Blancoe, Christopher Sgro, Tenielle Clinch, Mariana Melo, Alberto Pinzon-Charry, Anna Sullivan, Aideen McInerney-Leo, Jane Peake, Peter McNaughton, Tatiane Yanes

**Affiliations:** 1 Integrating Genomics into Medicine Group, Frazer Institute, The University of Queensland, Brisbane, Queensland Australia; 2 Department of Paediatrics, Faculty of Medicine, Dentistry and Health Sciences, The University of Melbourne, Melbourne, Victoria Australia; 3 Queensland Paediatric Immunology and Allergy Service, Children’s Health Queensland, Brisbane, Queensland Australia; 4 Faculty of Health, Medicine and Behavioural Sciences, The University of Queensland, Brisbane, Queensland Australia; 5 School of Environment and Science, Griffith University, Brisbane, Queensland Australia; 6 Department of Paediatrics and Child Health, The University of Queensland, Brisbane, Queensland Australia

**Keywords:** Immunogenetics, Genetics research

## Abstract

Genomic testing has become essential to diagnosing and managing paediatric inborn errors of immunity (IEI), necessitating the development of mainstream models of care to facilitate optimal delivery of testing. However, little is known about the experiences of families undergoing paediatric IEI genomic testing within mainstream settings and parental experiences with such conditions remain underexplored. Thus, this study aimed to describe the experiences of parents of children who underwent mainstreamed IEI genomic testing. Semi-structured interviews were conducted with 17 parents (14 mothers and 3 fathers) of children with an IEI and thematically analysed. Six themes captured (i) the diverse dimensions of distress related to paediatric IEI, (ii) the associated social, practical and financial implications, (iii) the involvement of children in their care, (iv) parental satisfaction with mainstreamed genomic testing, (v) the value of multidisciplinary care, and (vi) considerations surrounding genomic testing decision-making. Findings highlight the significant psychosocial impacts of paediatric IEI, including distinct social and emotional challenges. High satisfaction with mainstreamed IEI genomic testing was reported by all parents. Recommendations for improvement include developing tailored resources to address families ongoing psychoeducational needs, enhancing mental health support, and involving children appropriately. Collectively, these findings substantiate the benefits of mainstreamed IEI genomic testing, while expanding literature on the psychosocial impact of such paediatric conditions. Further exploration of families and children’s needs and development of tailored resources are essential to ensure the delivery of patient-centred care.

## Introduction

Inborn errors of immunity (IEI) refer to a group of severe genetic conditions that impact the immune system, increasing susceptibility to infection, autoimmunity, autoinflammation, allergies, malignancy, and/or bone marrow failure [1]. A total of 555 IEI have been described to date [1], with a combined prevalence between 1:1,000 to 1:5,000 [2]. These conditions are associated with causative variants in over 500 genes, with novel gene discovery ongoing [1]. IEI most commonly present in the paediatric setting [3], where they represent severe conditions associated with serious health complications and psychosocial challenges [4]. Whole exome sequencing (WES) is increasingly regarded as the most cost- and time-effective approach for molecular diagnosis of IEI [5]. Early molecular diagnosis can alter care and prompt initiation of targeted therapies, positively impacting clinical outcomes [5]. Specifically, a genomic diagnosis can result in precision therapies, such as biological drugs and curative treatment in the form of hematopoietic stem cell transplantation (HSCT) or gene therapy [6]. Recognizing the importance of early molecular diagnosis for paediatric IEI, genomic testing is increasingly integrated into routine care [7]. Optimal implementation of genomic testing requires a whole-of-system approach including multidisciplinary care, appropriate management of genomic and family health data, and considerations of the associated ethical, legal and social implications (ELSI) [8, 9]. To address these issues, new models of care have been developed to mainstream genomic testing into routine practice across multiple specialties, most commonly oncology [10].

To integrate genomic testing for paediatric IEI into primary care, the Queensland Paediatric Immunology and Allergy Service (QPIAS), Queensland Children’s Hospital, Australia, implemented and evaluated a mainstream model [11]. Briefly, key features included a genetic counsellor embedded in the department, monthly multidisciplinary team meetings, trio-WES sequencing as a front-line diagnostic test, and collaborative engagement with genetic pathology services [11]. Similar to other mainstream models [12], the QPIAS model was shown to improve access to, and uptake of genomic testing relative to the standard of care (i.e., referral to separate genetics services). Since January 2022, the service delivered genomic testing to an average of 36 patients annually. However, the experiences of families undergoing genomic testing for an IEI remain largely unexplored.

There is emerging evidence that parents of children with an IEI can experience distinct emotional challenges and adverse psychological outcomes [13]. Compared to parents of healthy children, parents of IEI children have reported increased anxiety, worry and substantial caregiver burden [14]. The most frequently indicated concerns among parents include the continuing nature of their child’s condition, ongoing care, fear of infection, and potential treatment side effects [14]. In particular, parents of children undergoing HSCT experience heightened parental distress, anxiety, and depression, especially when healthy siblings are involved in the donor process [15]. As IEI are lifelong conditions, understanding the challenges families face during, and beyond the genomic testing process is essential to delivering patient and family-centred care. Thus, this study aimed to explore i) the experiences of parents of children with an IEI and ii) parental experiences with IEI genomic testing via the QPIAS mainstream model of care.

## Methods

This study was approved by the Queensland Children’s Hospital Human Research Ethics Committee (HREC2023/102996) and University of Queensland Human Research Ethics Committee (HREC2024/HE000252).

### Study design and participant recruitment

Qualitative semi-structured interviews were selected to enable questions to be tailored to individual experiences and responses. Eligible participants were parents or legal carers (herein referred to as parents) of children who had IEI genomic testing through the QPIAS mainstream service from 2022 to 2024. QPIAS provides publicly funded allergy and immunology healthcare to Australian residents living in Queensland who are <18 years old. All families were eligible to participate regardless of genomic test result type or geographical location (i.e., regional or rural). Eligible individuals were identified and invited to participate in the study by members of the clinical team (authors T.Y., P.M., A.S., J.P., A.P.C., and M.M.). Purposive sampling was used to capture a diverse range of views across IEI conditions, demographic backgrounds and genomic test results. Prior to recruitment, participant characteristics (e.g., child’s IEI condition, genomic test outcome) were discussed to support representation across the cohort. Recruitment occurred separate to clinical care, with eligible participants contacted post genomic test result disclosure, with timing purposefully selected to minimise burden during periods of heightened emotional and medical needs. Recruitment continued until sufficient variation across participant responses had been captured to reflect the diversity of the population (i.e., IEI condition, genomic test outcome) and no new themes emerged. All families were provided with a copy of the study participant information form and written consent was obtained prior to the interview.

### Data collection

A semi-structured interview guide (Supplementary Material 1) was used to explore familial experiences with the IEI diagnostic process and the QPIAS mainstream service. After providing consent, participants took part in an interview conducted by A.C. (female research genetic counsellor with an education background), C.S. (male genetic counselling student), or T.Y. (female senior immunology genetic counsellor and researcher experienced in qualitative research) by phone or video conferencing. Researchers A.C. and C.S. initially observed T.Y. conduct an interview for training purposes, after which they completed interviews independently. Demographic and clinical information was collected via an online questionnaire administered prior to the interview.

### Data analysis

All interviews were audio-recorded, transcribed verbatim, and de-identified following transcription. Data was analysed using reflexive thematic analysis [16]. Initially, A.C. and T.Y. reviewed the first eight interview transcripts to familiarize themselves with the data. During the data familiarization process, the authors identified key features of the participants’ experience including diagnosis, treatment, and genomic testing for IEI, which were ascribed descriptive codes. Authors A.C. and T.Y. then discussed the descriptive codes, compiled a mind map to categorize codes and created preliminary themes and a codebook. The interview guide was adjusted at this stage, adding questions to explore emerging ideas (e.g., informational needs post-genomic testing) that arose during early data analysis, with additional recruitment (six parents) conducted to capture these concepts. An additional analysis was conducted following subsequent interviews, after which recruitment ceased as no new themes emerged. After completion of data collection, authors A.C., E.D. (female research genetic counsellor) and T.Y. applied an iterative, constant comparison approach using NVivo 12 software to analyse the remaining transcripts and reorganize the preliminary codes into final themes and subthemes. Data analysis continued through to the completion of the final manuscript. Any coding discrepancies between authors were identified whilst developing the codebook and resolved through ongoing discussion until consensus was reached.

As genetic counsellors, authors A.C., T.Y., E.D., and C.S. had substantial experience working with families affected by paediatric conditions. Additionally, both authors A.C. and T.Y. are mothers of young children. This positionality likely influenced the data collection, analysis and interpretation process. To enhance data transparency and accountability throughout the research process, reflexive journals documenting personal reflections, biases, and challenges were maintained. To ensure participant anonymity, all participants were assigned pseudonyms and genomic test outcome is described as diagnostic (i.e., pathogenic or likely pathogenic variant) or non-diagnostic (negative or variant of uncertain significance [VUS]).

## Results

### Participant characteristics

**Fig. 1:**
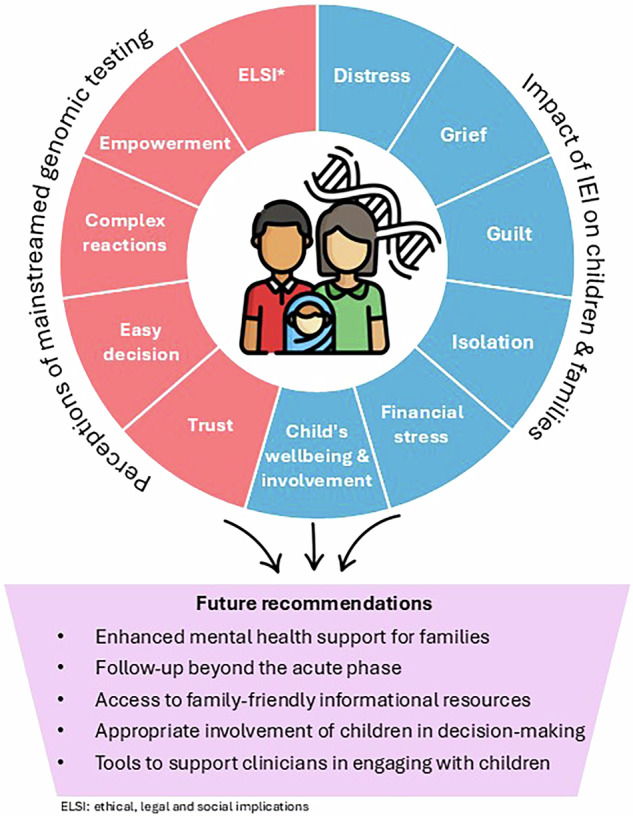
Parental experiences of paediatric IEI and mainstreamed genomic testing: key themes and future recommendations.

Of the 17 invited families, 14 consented to participate, comprising 17 parents (14 mothers and 3 fathers). Over half of parents lived in a metropolitan area (*n* = 10/17) and the mean age was 38 years (range 24–53 years). Children had a mean age of seven years at the time of genomic testing (range 3 months to 16 years). There were varied indications for genomic testing, including combined immunodeficiency, recurrent infections, aplastic anaemias, and autoinflammation. Over one-third of children (*n* = 6/14) received a molecular diagnosis and have since received tailored treatment in the form of HSCT or immunoglobulin replacement therapy (IRT). Of the 11 children who did not receive a diagnosis, one had a VUS and subsequently underwent genome sequencing due to remaining suspicion of an underlying monogenic condition. Interviews were conducted on average nine months after genomic test result disclosure (range 2 to 18 months). Interviews ranged from 24 to 90 minutes (mean=54 minutes). Responses were categorized into themes and subthemes under two main domains: i) impact of IEI on children and families and ii) perceptions of mainstreamed genomic testing. Representative quotes for each domain are shown in Tables 1 and 2, with themes summarised in Fig. 1.

**Table 1:** Domain 1 representative quotes.

Theme/subtheme	Representative quote
*Diverse dimensions of distress*	
Initial and sustained trauma	*“A very traumatic night* [Bonnie’s initial hospitalization]*. It’s up there. You know, I’ll remember that like the night my dad died…”* **(Mother of Bonnie, 8-month-old, diagnostic result)** *“*[participant crying] …*it doesn’t matter how many times you say it* [child’s medical journey]*, it still just happens to trigger that emotional reaction.”* **(Mother of Indiana, 4-month-old, diagnostic result)**.
Grief	*“You’ll be grieving the future of what you had in your head at that time…that was probably the biggest thing … it’s not just processing what’s going to happen, but it’s also if you had an idea in your head about what that child’s life looked like or just simple things like what Christmas is going to look like. That can all change and sometimes there’s a lot of grief that goes along with that*.” **(Mother of Indiana, 4-month-old, diagnostic result)**
Guilt	*“Definite guilt from our point of view of* [Frances’ father] *and I. But as lots of people have told us, like it’s not our fault and then it’s not our parents’ fault… but I mean, we’ll forever feel that.”* (**Mother of Frances, 11 years old, diagnostic result)** *“…whether rightly or wrongly I feel like a failure, you feel like you second guess absolutely everything you’ve done, I second guessed everything, even in pregnancy… And then the thought of it being a genetic thing, it made me feel guilty as well.”* **(Mother of Samuel, 8-months-old, non-diagnostic result)** *“I’ve got this whole bated breath about what’s happening with my* sister’s *daughters* [undergoing carrier testing] *… I’m really praying that they don’t have it* [familial variant] *and they’re not carriers… at the time I’m celebrating, could be their grief time. … it just draws it out and rather than just closing it and then you feel like you’ve got all the experience to be an emotional support to them and I’m just back in it then.”* **(Mother of Max, 3 years old, diagnostic result)**
*The ripple effect*	
Social Isolation	*“… we’re quite social, a lot of friends, so we had to stop doing that for quite a while. Obviously, we couldn’t go to our friends’ barbecues and social events so that was non-existent for quite a while …”* (**Mother of Frances, 11 years old, diagnostic result)** *“It was a huge disruption* [relocation from regional town]*, it was a huge stress…my husband was still trying to work nine to five at that point as well…my husband was not coping with us being gone for that length of time and not knowing when we’re coming back…”* **(Mother of Samuel, 10-months-old, non-diagnostic result)** *“There’s a lot more planning on the process* [of doing an activity] *we can’t just go for a long weekend or let’s go and do something. We have to plan when his treatment’s going to be… We have to think about*, [do] *we take stuff with him or do we go to the hospital…you just don’t know about it until you email the hospital…”*(**Mother of Theo, 6 years old, non-diagnostic result)**
Impact on family dynamics	*“No I don’t want tell anyone* [carrier status]*, I think it was mostly because I didn’t want the grandparents to blame- to play the blame-game.”* **(Mother of Indiana, 4-month-old, diagnostic result)**.
Financial impact	*“Financially it’s been tough. We buy her special formula and her medication, hospital parking*…” **(Mother of Indiana, 4-months-old, diagnostic result**) *“…we probably have ended up borrowing quite a bit of money just for health, that’s a big part of our life.”* **(Mother of Clementine, 12 years old, diagnostic result)**
Social and psychological impact for child	*“It’s* [IEI] *really made* her *development quite slow…* she has *missed a heap of school… and especially with COVID like the whole year they didn’t go* [to school]*”* **(Mother of Clementine, 12 years old, diagnostic result)** “*We don’t want to highlight it to kids at school that he’s got issues because obviously then kids start picking on and making issues of it*…. *And socially that seems to be more impactful* [for Theo] *than the immune side of things…***(Mother of Theo, 6 years old, non-diagnostic result)** *“* [Frances] *has changed a lot in the way of friendship circles…obviously she is not at school, so having that and then just friends, just that contact with friends and just not being able to be social*.” **(Mother of Frances, 11 years old, diagnostic result)** *“To do some of the sessions* [IRT] *we got to a point we basically had to pin him* [Theo] *down to do it.”* **(Father of Theo, 6 years old, non-diagnostic result)**
Seeking child psychosocial support	*“So I was wanting to explore if we should be seeing a psychologist for him just to make sure there’s no long-term mental issues from doing something so different. But it hasn’t been too successful so far*.” **(Father of Theo, 6 years old, non-diagnostic)**
Information needs	*“A sourcing of information about your condition that is correct and just look here for what you need, that would be helpful…and we did get that to an extent, but something as simple as what’s the life expectancy? I don’t think we got that. I was finding that out from medical journal… but because you have these kinds of questions, which then you spiral…and you just end up Googling…* **(Father of Max, 3 years old, diagnostic result)** *“I think the hardest thing is the uncertainty with the whole thing. There are no answers, and you have no idea of the outcome*. **(Father of Theo, 6 years old, non-diagnostic result)** *“…But I met so many people during that time* [child’s hospitalization] …*I was given lots of information, big folders, big books. And I know that everyone had their job to do, but it was too much…”* **(Mother of Meg, 12 years old, non-diagnostic result)**
Balancing children’s presence and participation	
Appropriate involvement of children	*“I would say she was involved* [decision for genomic testing], *but she didn’t understand what she was doing”* **(Mother of Hermione, 12 years old, diagnostic result)** *“We’re really happy with really with our doctors, but they don’t necessarily explain things to the kids well… no one’s really ever explained to* him *what’s going on.”* (**Mother of Theo, 6 years old, non-diagnostic result)** *“… every appointment we have to have* [child] *with us, so it doesn’t leave much room to then ask questions. And then I don’t want to ask some of them in front of him because he listens*. He *hear(s) everything. [They] may not be watching and watching the conversation happen, but he’s listening to everything, and he’ll ask you a question.”* (**Mother of Theo, 6 years old, non-diagnostic result)**

*IRT* Immunoglobulin Replacement Therapy,

*IEI* Inborn Error of Immunity.

**Table 2:** Domain 2 representative quotes.

Theme/subtheme	Representative quote
The value of mainstreamed genomic testing	
Grateful for timely genomic testing	*“Fantastic. It’s a godsend* [mainstreamed genomic testing] … *I don’t think…we’d be where we are now without that being offered to us, without that clarity and treatment and all being provided.”* **(Father of Jack, 1 year old, diagnostic result)** *“I think it’s* [mainstreamed genomic testing] *great, I think it’s wonderful that we live in this day and age that it can be done, and I think that it’s really helpful in certain situations”* **(Mother of Samuel, 8-months-old, non-diagnostic result)**
Diagnostic results are empowering	*“… in the future when we do want to have more children, we can do what we can to prevent those future children from having the same genetic condition* **(Mother of Anthony, 3 months old, diagnostic result)** ***“…****very positive, uplifting impact. Really fantastic…I think by the time we had received that diagnosis, that, you know, “Hey, this is curative in nature”* **(Father of Jack, 1 year old, diagnostic result)**
Diverse reactions to non-diagnostic results	*“We didn’t get any answers, c’est la vie. It didn’t faze me. If we got it I would have been of the same opinion.”* **(Mother of Joe, 16 years old, non-diagnostic result)** *“In one way it’s a little bit frustrating it’s like I just want this to be finished, I want answers.”* **(Mother of Lily, 8 years old, non-diagnostic result)** *“Oh, it was a relief. I mean it was a relief that it was certainly a relief that there was no genetic cause”* **(Mother of Meg, 12 year old, non-diagnostic result)**
Multidisciplinary teams are key to successful mainstreaming	
Trust and faith in medical team	*“All the doctors in his team now have been a part of this team since he was three years old. Yeah amazing, amazing, amazing team doctors here in Queensland”*. **(Mother of Joe, 16 years old, non-diagnostic result)** “*…We found it easy to trust them* [medical team]*…We continuously would say that to* [Frances] *over and over, we trust the doctor, we trust them*…. I *guess, the openness, not just the benefits of what’s going to happen, but being told what might go wrong …not just, putting rose-coloured glasses on us to see it’s all going to be OK…We knew what we were getting ourselves into* [consenting for genomic testing].” **(Mother of Frances, 11 years old, diagnostic result)**
Genetic counsellor as an added bonus	*“It was pretty mysterious going into it, like, really a genetic counsellor, why do I need this? …I Googled it, ‘what kind of questions should I ask a genetics counsellor?’ Because somebody had impressed upon me the importance. I knew it was important, but didn’t really know what it was all about.”* **(Mother of Bonnie, 8-months-old, diagnostic result)** **“***My advice is any kind of genetic testing on children and especially small children the genetic counselling should come before the genetic testing*” **(Mother of Joe, 16 years old, non-diagnostic result)** “*It’s a very different type of counselling* [genetic counselling] *…to what’s provided by the social worker…because of where family is in their journey…. I think around the ‘now-ish’ time is probably not such a bad time for* [genetic counsellor] *to have another session*” **(Father of Max, 3 years old, diagnostic result)**
*Genomic testing decision making: why, when and how*	
Easy decision	*“The genetic testing to us was just a given. Like we were never going the question that it wasn’t going to hurt anyone*…” **(Mother of Frances, 11 years old, diagnostic result)** *“…it wasn’t like it was a high-pressure thing* [consenting to genomic testing]*, every other decision that we made with* [child] *was far more stressful and pressured…. it wasn’t a traumatizing experience.”* **(Mother of Samuel, 8=months-old, non-diagnostic result)** *“I get that there’s all these ethical dilemmas… but to me it was a very easy decision* [consenting to genomic testing] *…. It’s much better to make an informed decision than not….thank you for doing all your due diligence and showing me the paperwork, but yes, I’m going to do that.”* **(Father of Henry, 1 year old, diagnostic result)**
Complexities with genomic testing decisions	*“I think my biggest concern at the time was we were taking that choice away from* [Bonnie]*. Obviously, them being so young, they don’t have that voice to say, ‘actually, I don’t want to find out’. And it was just taking that, making that decision for them. I think I was a bit uneasy about.”* **(Mother of Bonnie, 8-months-old, diagnostic result)** *“I do remember now that we spoke to a genetic counsellor, and I guess tried to understand what the potential implications in the future with insurance are, with life insurance, travel insurance of these type of things”* **(Father of Thoe, 6 years old, non-diagnostic result)**
A time and a place	*“At the time of the appointments…it was very overwhelming. I had a lot of the questions that I didn’t get answered at the time or things like that because it was so much to take in all at once it was… being by myself at that stage as well, it was a lot to process at the time…”* **(Mother of Lily, 8 years old, non-diagnostic result)** **“***you’re in and out of hospital … And there’s only so much information that any person can take on and really, truly understand”* (**Mother of Frances 11 years old, diagnostic result)** *“I don’t specifically remember consenting to the genetic testing because it kind of got lost in amongst all these other research studies that we were being given”* **(Mother of Indiana, 4-month-old, diagnostic result)**

*IEI* Inborn errors of immunity.

### Domain 1: Impact of IEI on children and families

#### Theme 1: Diverse dimensions of distress

Almost all parents described experiencing distress and trauma in the acute phase of illness, which stemmed from their child’s medical emergencies, prolonged hospitalizations, and subsequent complex treatments (i.e., HSCT and IRT). Such feelings were ongoing, and in some cases, presented as post-traumatic stress symptoms, including frequent memories of these events, avoidance of hospital settings, and fear of recurrence. Events such as medical procedures, ongoing treatments, and familial risk discussions often triggered such feelings among many parents. Parents expressed a strong desire to prevent further pain or trauma for their children. Grief was also noted among several participants, which often stemmed from changes to the expected future life of their child. Additionally, feelings of guilt were reported, especially among parents who are carriers of their child’s condition. Such feelings of grief and genetic responsibility often extended to participants’ other children, family members, and possible future children. Prolonged cascade testing often extended parental anguish as they anxiously awaited family test results instead of celebrating their child’s recovery.

#### Theme 2: The ripple effect

Parents described numerous ongoing challenges distinct to IEI across multiple aspects of their lives, including the impact of social isolation, concerns for their child’s psychosocial wellbeing, and challenges in managing their condition with limited information and resources. Feelings of social isolation were frequently reported by parents due to the nature of IEI, which required many to physically isolate for extended periods. In some cases, families were required to remove siblings from daycare and minimize social interactions. High-risk infection periods (e.g., influenza seasons and the COVID-19 pandemic) further exacerbated fear of infection and prolonged social isolation for families. Subsequently, parents referred to feeling emotionally and socially isolated from their support systems. Such feelings were intensified among families from regional and rural areas that required relocation to metropolitan hospitals for treatment. These families faced additional stressors, such as a lack of local community support and needing to arrange alternate care for siblings. Lastly, families frequently reported facing substantial financial stress due to increased expenses, such as treatment costs, travel and hospital parking, and reduced capacity for employment particularly whilst physically displaced.

Concerns about the social and psychological impact of living with an IEI for children were raised by all parents. The impact of extended periods of isolation and school absences were of particular concern, which were thought to have affected their child’s education and social development. A few parents noted their child’s reluctance to discuss their health with friends, family and in school settings. These feelings were thought to be driven by not wanting to be seen as different from peers and feeling embarrassed to discuss their health needs. Furthermore, parents described children being “*forced to mature”* (Mother of Grayson, 15 years old, non-diagnostic result), due to facing challenges such as life-threatening events, invasive procedures, and involvement in healthcare decisions. However, parents recognized that such concerns were reflective of their observations and there may be discordance between their perceptions and those of their child. Furthermore, some parents noted that ongoing medical treatments had resulted in their child having considerable medical related concerns, such as needle phobias and fear of hospitals. Finally, parents expressed a need to explore psychosocial support for their child amidst ongoing worry about the impact of their condition on their wellbeing. One family reported seeking ongoing psychological support for their child, however they found it difficult to coordinate with other care commitments and school attendance.

Parents frequently described experiencing ongoing information needs, which intensified the challenges in managing the ongoing effects of their child’s condition. Informational needs transcended the outcome of genomic testing and included questions about their child’s health condition, treatment options, prognosis and genetic implications. Parents reflected on challenges accessing patient-friendly resources that provided such information and having to seek answers online. While parents acknowledged that informational resources had been provided, there was often a disconnect between the information they sought and what was given. Evolving informational needs were also noted as families transitioned beyond the initial acute phase and their child’s developmental needs changed. An easily accessible online repository of information was suggested by some.

#### Theme 3: Balancing children’s presence and participation

Appropriate involvement of children in medical decision-making and conversations was consistently reported as limited, with many parents describing challenges in determining when and how to include their child. There was no support or guidelines to help parents communicate with their child, leaving parents feeling responsible but ill-equipped to have conversations with their child about their condition. Parents expressed a desire to have their child more involved in decisions related to their healthcare. Most parents reported that the medical team rarely involved children in discussions using child-friendly explanations, which was desired by parents. Particularly, engagement with older children in the genomic testing process varied, with some parents questioning their child’s level of understanding. Select parents expressed concerns about making decisions on behalf of their child, such as genomic testing and respecting their child’s autonomy. Parents also noted the importance of tailoring medical conversations with children to their maturity and developmental stage accordingly. Lastly, the option to have child-free appointments was considered valuable, allowing parents to focus on and discuss sensitive topics, such as life expectancy and future challenges, which they found difficult to address in the presence of their child.

### Domain 2: Perceptions of the mainstreamed genomic testing process

#### Theme 4: Significance of mainstreamed genomic testing

Regardless of genomic test results, all parents were grateful for timely access to genomic testing through the mainstream model of care. Continuity of care and direct access to genomic testing without the need for additional referral to external services was especially valued. In fact, all parents viewed genomic testing as part of their child’s continuum of care, spanning from initial presentation to diagnosis and subsequent treatment. Most parents also found it difficult to distinguish between genomic testing and their child’s hospitalizations.

Parents found receiving a genomic result useful, even if it did not result in a clear diagnosis. Specifically, parents of children with positive results highlighted the value of receiving diagnostic clarity and much sought-after answers for their child’s health condition. For these families, a molecular diagnosis resulted in changes to their child’s management and treatment and informed future reproductive planning for themselves, their child, and their extended family. Parents of children who received a non-diagnostic result still considered the knowledge valuable as it provided some families with relief, especially when considering the familial implications of a genetic diagnosis. Other responses were more nuanced, with some parents feeling frustrated at a lack of answers, while others were accepting that they may never know the cause of their child’s condition. In one case, a parent erroneously inferred that a non-diagnostic result meant that their child’s condition was not genetic.

#### Theme 5: Multidisciplinary teams are key to successful mainstreaming

High satisfaction with the genomic testing process was further driven by parents trust in their child’s medical team. For some, these relationships had been established over several years of sustained healthcare for their child. Parents reported that the medical team were transparent about the benefits and risks of procedures, testing and treatments, and gave them time to reflect on their motivations for pursuing genomic testing and managed their expectations well. These factors led to high trust in the clinical team.

Most parents reported being initially unfamiliar with the role of a genetic counsellor and the purpose of genetic counselling. However, almost all parents recalled the significant positive contribution a genetic counsellor added to their child’s medical team. Parents indicated that genetic counselling appointments allowed them to gain a clearer understanding of the heritability of the condition, family planning considerations, data privacy considerations and possible insurance implications. Furthermore, some parents who were carriers of their child’s condition highlighted the emotional support provided by genetic counsellors and valued the opportunity for follow-up as their needs evolved.

#### Theme 6: Genomic testing decision making: why, when and how

For most parents, the decision to consent to genomic testing was easy. Testing was viewed as an opportunity to provide a diagnosis and a clear treatment pathway. Some parents reported that the decision for their child to undergo genomic testing was easy relative to other medical choices (e.g., HSCT). Given that many parents had witnessed their children suffer life-threatening medical emergencies and invasive procedures, parents expressed relief that genomic testing was non-invasive compared to other medical investigations. The opportunity to engage with a genetic counsellor embedded within the service was valued by these parents. Nevertheless, several parents felt overwhelmed by genomic testing-related information, particularly when such discussions coincided with traumatic events and needing to provide consent for multiple procedures, genomic testing, and treatment. Receiving information without a support person present was also noted as challenging, placing the knowledge burden on the single carer. For one parent such feelings of being overwhelmed were further exacerbated by receiving invitations from several research studies. While genomic testing was an easy decision for most parents, select participants expressed concerns about respecting their child’s autonomy, ensuring data privacy and mitigating possible discrimination.

## Discussion

This study provides a nuanced understanding of the experiences of families with paediatric IEI and mainstreamed genomic testing. Findings demonstrate the extensive emotional and psychosocial impact of such childhood conditions. While findings align with previously reported experiences in paediatrics rare diseases [17, 18], our study reveals the unique constellations of impacts associated with IEI, including the social and emotional isolation and ongoing fear of infection. The large geographical area serviced by QPIAS amplified such concerns and limited immediate support networks for families from regional and rural areas [19], which is similarly reported in paediatric oncology settings [20]. Given that genomic testing was offered at point of care, parents viewed testing as part of routine care and often found it difficult to distinguish from other investigations, particularly when delivered in the acute phase of illness [21, 22]. The strong connection and trust in the medical team, combined with access to timely genomic testing, inspired high parental satisfaction with mainstreamed genomic testing. Nevertheless, areas for improvement were identified, including the need to address the ongoing psychoeducational needs of parents and children.

The psychological impact of chronic childhood conditions on both affected children and their families is well reported, including for immunology conditions such as for severe combined immune deficiency (SCID) [17, 23, 24]. Within this study and the context of genomic testing, families described sustained feelings of distress and grief. Some parents also expressed salient feelings of frustration when genomic testing did not provide a causal explanation for their child’s condition, a response similarly documented in the rare disease literature [25]. Feelings of transmission guilt were also commonly described, especially among parents who were carriers for their child’s condition [26]. Moreover, due to the shared nature of genomic variants, feelings of guilt were often exacerbated by the possibility of having transmitted the condition to other children and family members. This extended genetic burden is consistent with the wider literature on paediatric genomic testing [26–28]. These findings highlight the complex emotional landscape that families navigate during the immediate acute phase of illness, as well as the psychological sequalae [29]. It is important that clinicians are attuned to the psychological needs of families and identify when they may require additional mental health support. Despite such needs, access to parental and family mental health services varies across services, and in many cases are sparsely available.

The social and emotional impact of chronic illnesses in childhood is increasingly being explored. Previous research has identified lower self-esteem and resilience, diminished health-related quality of life, and higher levels of emotional difficulties among children who experience rare conditions [30, 31]. Children not involved in age-appropriate decision-making or provided with tailored information commonly express frustration, report not feeling heard, and poor understanding of their condition [32]. Consequently, they can have reduced treatment adherence and disengage with health service post-transitioning to adult care [33, 34]. While such issues are areas of active research in paediatric oncology, there has been little exploration in paediatric immunology settings. As children’s experiences are often inferred from studies focused on parental perceptions, there is a critical need for research that directly captures children’s experiences living with an IEI, undergoing genomic testing, and their related support needs. Understanding the experiences and needs of paediatric patients related to IEI conditions and genomic testing will enable clinicians to better support families and guide the development of resources that address their unmet needs.

Our findings support the increasing evidence for mainstreaming genomic testing via new models of care that consider the associated testing and genetic counselling implications. Parental trust and connection with their child’s medical team was a key driver in their decision-making and satisfaction with genomic testing [18]. As previously reported [35], mainstreaming was further valued as it eliminated the need for additional referrals and appointments. However, the multidisciplinary aspect of the model of care, which included access to genetic counselling aided families who expressed concerns with genomic testing decision-making. Commonly reported reasons for parents declining genomic testing can include fear of future genetic discrimination, feeling overwhelmed, and the desire to defer testing until the child is older and has the capacity to consent [36, 37]. With the increasing utility of genomic testing for IEI, it is important that clinical immunologists are aware of the unique considerations of paediatric genomic testing to better facilitate timely informed decision-making, respond to family needs, and identify when additional support is needed. Resources have been developed to support mainstream consent, including patient-friendly fact sheets, animations, and tailored genomics education for clinicans [38–41].

There is an increasing necessity to mainstream genomic testing into specialist clinics, with diverse models and strategies assessed [12, 42–45]. Thus, this study has broader implications beyond paediatric IEI. Key to the success of this model included i) embedding of an experienced genetic counsellor (author T.Y.) who was familiar with multiple care settings and coordinated service improvements [42, 44, 45], ii) an immunogenetics champion (clinical immunologist, author P.M.) [46], iii) hospital executive level endorsement, and iv) multidisciplinary collaboration, including with pathology services [12]. Collectively such factors supported mutual learning, provision of holistic patient care, and service improvements. While the establishment and previous evaluation of this model was largely conducted intuitively [11, 47], future research and the development of new models of care should incorporate implementation science principles from the outset. Such approaches will enhance the rigour of future research and support the integration of genomic testing into diverse clinical settings effectively and equitably [47].

These findings substantiate the recommendations from Pursey et al. [23] in relation to supporting families of children diagnosed with SCID, including improved access to family education, mental health care, telehealth appointments outside of the acute inpatient phase, and provision of reputable informational resources. Our study further extends these findings via the inclusion of a broader spectrum of IEI conditions and parents of older children during the genomic testing process. While IEI are heterogenous, our study highlighted common experiences across conditions and genomic test result outcomes, which can inform future recommendations for this population. Additional recommendations include the opportunity for child-free appointments with parents to allow for discussion of sensitive topics and development of disease-specific resources. Empowering understanding among paediatric patients and their families requires the development of age-appropriate informational resources (e.g., fact sheets, videos, story books for younger children) [9, 48, 49] alongside tools that support clinicians to feel competent engaging children of various ages in their care. Incorporation of youth-friendly genetic counselling frameworks should also be considered [50].

Findings should be interpreted in line with the limitations of this study. Firstly, our findings indicate that genomic testing is intertwined with the impact of the IEI diagnosis and treatment. Therefore, the results should be understood within the broader context of families adapting to the challenges of an IEI diagnosis and ongoing care. The cohort primarily consisted of mothers and no children were included in the study. Thus, future research should aim to involve more fathers, children, and possibly siblings and extended family members as their experience may differ. Recruitment also occurred 2 to 18 months post-genomic test result disclosure; while this approach may have influenced participants recollection and responses, it was employed to avoid contacting families during periods of acute illness and to minimize participant burden. Nevertheless, a key strength of the study is the diverse perspectives of parents of children with a broad range of IEI, genomic test result types, and of various age groups. Overall, our findings provide valuable evidence that a mainstream model of care for paediatric IEI genomic testing is highly acceptable to parents. However, gaps in care remain, and families continue to experience substantial emotional challenges and report ongoing informational and emotional needs. As genomic testing becomes increasingly integral to IEI paediatric care, it is imperative that mainstream services are equipped to adequately address the distinct testing and counselling needs of families.

## Supplementary information

Supplementary Material 1

## Data Availability

The data that support the findings of this study are available on request from the corresponding author and subject to ethical approval.
