# Supplementary material for: “It’s a Godsend”: Parental experiences of genomic testing for paediatric inborn errors of immunity
Source: Eur J Hum Genet. 2025 Jul 29;33(10):1342–9. doi: 10.1038/s41431-025-01917-7 (PMC12479962; doi:10.1038/s41431-025-01917-7)
Supplement: Supplementary file 1 — Supplementary Material 1 [file 41431_2025_1917_MOESM1_ESM.docx]

**Introductory Script**

- Thank participant for agreeing to take part in this study.
- Briefly introduce the researcher and brief professional background.
- Introduce the study and briefly discuss its purpose.
- Reassure participants that they are free to talk about any aspect of their experience or attitudes. There are no right or wrong or even typical answers to any of the questions that we will discuss. All views will be treated with equal relevance to the research.
- Remind participants that, with their permission, the session/interview will be recorded.
- Reassure confidentiality and the participant’s right to leave or stop the interview at any time.
- Clarify that the interview will take 30min – 1 hour.
- Ask whether participants have questions before commencing.

Date of interview:

Interviewer:

Participant ID:

[**Notes:**__________________________________________________________________________________________________________________________________________________________________________________________________________________________________________________________________________________________________________________________________________](Notes:____________________________________________________________________________________________________________________________________________________________________________________________________________________________________________________________________________________________________________________________________________)

| Topic | Key Question | Prompts |
| --- | --- | --- |
| Background | 1. Can you tell me a bit about yourself and your family and children? | - - - - *Job*       - *Number of children*       - *Family background* |
| Experience caring for a child with IEI | 1. What impact has this condition had on your family? 2. What impact has this condition had on your child themselves? | - - - - *How old was your child when they first became ill?*       - *Work/school disruption?*       - *Effects on other children?*       - *Displacement and travel?*       - *Financial burden?*       - *Plans for the future?*       - *Social impact?* |
| Experience with genomic testing | 1. Can you please describe how your child came to have genomic testing? 2. When making the decision to have genomic testing, what sort of questions or concerns (if any), if any, did you have about test? 3. What involvement did your child have in this consent process? (If relevant to age). | - *Who first raised genomic testing?* - *Information: potential results, insurance, risk to family members? Written and consent form?* - *Expectations: diagnosis, changes to care or management changes, reducing uncertainty?* - *Questions: how the sample would be collected, turn around time, insurance, risks?* - *Does your child understand why the testing was being conducted? Was this important?* |
| Informed consent | 1. One of the things we’re interested in is whether people are making “informed decisions” when having genomic testing. What does an informed decision mean to you? 2. Do you feel you made an informed decision about your child’s genomic testing? Please explain. 3. What information was most important for you when making your decision about genomic testing? 4. How would have liked to have received this information/in what format? | - *Meaning informed consent: given enough time, information, understand risk/benefits?* - *Potential barriers to consent for child: stress of child’s illness, too much information, information too complex?* - *Potential strategies to support consent: Trust/relationship with the medical team, written information, videos?* - *Was there any further information you would have liked to have received?* |
| Response to results | 1. What is your reflection of how your results were returned? 2. What support, if any, have you received after receiving your child’s genomic testing results? 3. Are there supports or resources that you think could assist families when they receive the result? 4. What impact has the result had for your child and their future 5. What has been the overall impact of your child’s genomic test results on your immediate family?  - What about your wider family members (first and second degree) relatives? | - *Possible results: positive, negative, variant of uncertain significance* - If no - did you feel that you needed any support during this process - *Impact on child’s care: none, transplant, infusions, other?* - *Feelings: sadness, guilt, anxiety, frustration, relief, hopeful?* - *Is there any additional support you felt was needed*? - *Uncertainties related to results, child’s healthcare, risk to family members, family planning.* - *Family: risk to relatives, coping with diagnosis, family planning, sharing/communicating information and results with family.* |
| Overall thoughts | 1. Do you have any suggestions for improving the genetic testing process when considering what is or would have been best for your family? 2. Are there any further comments you would like to add or suggestions would like to make about your experience with genomic testing? |  |
